# Challenges of Quantifying PRISM 2.0 and Tablet Use

**DOI:** 10.1093/geroni/igab046.1195

**Published:** 2021-12-17

**Authors:** Walter Boot, Neil Charness, Jerad Moxley

**Affiliations:** 1 Florida State University, Tallahassee, Florida, United States; 2 Weill Cornell Medicine, New York, New York, United States

## Abstract

As with the PRISM 1.0 trial, an important outcome of the PRISM 2.0 trial is use of the PRISM system and use of the PRISM system compared to the control condition (a standard tablet without the PRISM software). Frequent use over time is an important measure of system success. Further, use data provide key measures of system usefulness and usability. What features do participants use most and how often? Within those features, what activities do they engage in? What are the patterns of use throughout the trial, and how does PRISM system use compare to the control condition? However, quantifying use is not an easy task. This talk presents the challenges of quantifying use of a complex, multi-faceted system, and of making meaningful comparisons in use between two very different systems. Analysis approaches and solutions are discussed.

